# Automated Scoring of Tablet-Administered Expressive Language Tests

**DOI:** 10.3389/fpsyg.2021.668401

**Published:** 2021-07-22

**Authors:** Robert Gale, Julie Bird, Yiyi Wang, Jan van Santen, Emily Prud'hommeaux, Jill Dolata, Meysam Asgari

**Affiliations:** ^1^Center for Spoken Language Understanding, Oregon Health & Science University (OHSU), Portland, OR, United States; ^2^Boston College, Chestnut Hill, MA, United States

**Keywords:** speech, expressive language, language disorders, assessment, automated scoring, neural language models

## Abstract

Speech and language impairments are common pediatric conditions, with as many as 10% of children experiencing one or both at some point during development. Expressive language disorders in particular often go undiagnosed, underscoring the immediate need for assessments of expressive language that can be administered and scored reliably and objectively. In this paper, we present a set of highly accurate computational models for automatically scoring several common expressive language tasks. In our assessment framework, instructions and stimuli are presented to the child on a tablet computer, which records the child's responses in real time, while a clinician controls the pace and presentation of the tasks using a second tablet. The recorded responses for four distinct expressive language tasks (expressive vocabulary, word structure, recalling sentences, and formulated sentences) are then scored using traditional paper-and-pencil scoring and using machine learning methods relying on a deep neural network-based language representation model. All four tasks can be scored automatically from both clean and verbatim speech transcripts with very high accuracy at the item level (83−99%). In addition, these automated scores correlate strongly and significantly (ρ = 0.76–0.99, *p* < 0.001) with manual item-level, raw, and scaled scores. These results point to the utility and potential of automated computationally-driven methods of both administering and scoring expressive language tasks for pediatric developmental language evaluation.

## 1. Introduction

Untreated and undiagnosed developmental language disorder (DLD) is prevalent in young children (Tomblin et al., 1997; Conti-Ramsden et al., 2006; Grimm and Schulz, 2014; Rosenbaum and Simon, 2016) and can have serious behavioral and educational consequences (Clegg et al., 2005). Wide-reaching language assessment is urgently needed not only for early identification of DLD but also for planning interventions and tracking the efficacy of these interventions. Such efforts, however, add strain to scarce and overtaxed clinical resources. To address this challenge, clinicians, educators, and researchers have begun to explore alternatives to standard assessment paradigms that can be more easily and more reliably administered and scored.

Conventional language test administration and scoring is a labor-intensive and time-consuming task relying on significant clinical expertise. Assessment is typically conducted during a clinical visit by a speech language pathologist (SLP) using a battery of standardized language tests in addition to criterion-referenced and dynamic measures. Responses are scored using paper score sheets, which are marked in real time and then later reviewed by the clinician. In addition to the practical overhead required to administer such assessments, the scoring of these tests can suffer from intra- and inter-rater variability (Denman et al., 2017). Although some progress has been made in developing automatic assessments for receptive language (i.e., language comprehension), there is no automatic assessment that elicits and analyzes spoken responses to expressive language tasks (Marble-Flint et al., 2019, 2020). A sizeable fraction of children with DLD have primary difficulty with expressive communication (Tomblin et al., 1997). The nature of an individual's language disorder impacts etiology, intervention, and psychiatric sequelae (Boyle et al., 2010; Yew and O'Kearney, 2013). Given that computerized administration ought to mimic conventional administration, spoken responses must be used in any proposed computerized assessment, just as they are in many subtests that contribute to general language composite scores (e.g., Semel et al., 2003). While language assessment for children has predominantly utilized conventional face-to-face administration, automated testing could increase clinician efficiency, access to services, standardization of administration and scoring, and even child interest (García Laborda, 2007; Noland, 2017).

In this paper, we investigate the utility of a computerized tablet-based child language assessment instrument, modeled after the Clinical Evaluation of Language Fundamentals (CELF-4) (Semel et al., 2003). Using child language data collected both with this computerized instrument and with standard paper-and-pencil administration, we demonstrate the accuracy and feasibility of an automated scoring system for four expressive language tasks. The scoring methodology fine-tunes representation learning models to predict the score for responses to individual test stimuli, from which raw and scaled scores can be derived. In contrast to models that take manually-crafted measures of language as input, our deep neural network (DNN)-based model directly estimates scores directly from the transcripts. We find that our computerized scoring system yields very high item-level accuracy and summary score correlations when applied to both clean and verbatim speech transcripts. These results demonstrate the promise of computerized approaches to scoring expressive language tasks, which in turn can support clinicians tasked with diagnosis and extend the reach of services for children with developmental language disorder.

## 2. Materials and Methods

### 2.1. Data

#### 2.1.1. Participants

Participants in this analysis include 107 English-speaking children aged 5–9 years. These children represent the subset of participants from our larger study who completed automatic test administrations. Our participants and their families provided informed consent, according to our institutional review board policies. The children's demographic information is presented in Table 1. In order to ensure the utility of our system for a diversity of child ability, participants included children with Autism Spectrum Disorder (ASD, *n* = 20), Attention-Deficit Hyperactivity Disorder (ADHD, *n* = 19), Developmental Language Disorder (DLD, *n* = 22), and typical development (TD, *n* = 46). Diagnoses were confirmed via a combination of parent report of medical diagnosis, parent report of special education eligibility criteria, and expert review of parent-provided developmental history.

**Table 1:** Baseline characteristics of participants.

		**All (*N* = 107)**	**TD (*N* = 46)**	**ADHD (*N* = 19)**	**DLD (*N* = 22)**	**ASD (*N* = 20)**
	Female/Male	44%/56%	57%/43%	53%/47%	36%/64%	15%/85%
	Age in years, X (SD)	7.29 (1.05)	7.03 (1.13)	7.54 (1.18)	7.54 (0.75)	7.38 (0.97)
**Race**						
	Asian, N (%)	3 (3%)				3 (15%)
	Black/African American, N (%)	3 (3%)		1 (5%)		2 (10%)
	White/Caucasian, N (%)	90 (84%)	43 (93%)	15 (79%)	20 (91%)	12 (60%)
	More than one race, N (%)	11 (10%)	3 (7%)	3 (16%)	2 (9%)	3 (15%)
**Ethnicity**						
	Hispanic/Latino, N (%)	10 (9%)	2 (4%)	4 (21%)	1 (5%)	3 (15%)
	Not hispanic/Latino, N (%)	97 (91%)	44 (96%)	15 (79%)	21 (95%)	17 (85%)
**Language scores**						
	EV scaled, X (SD)	12.48 (2.59)	13.59 (1.90)	12.89 (2.02)	10.82 (2.99)	11.35 (2.70)
	FS scaled, X (SD)	11.08 (3.67)	12.70 (2.29)	11.00 (2.36)	10.09 (3.34)	8.55 (5.58)
	RS scaled, X (SD)	10.62 (3.84)	12.65 (2.70)	10.42 (2.04)	8.32 (3.72)	8.65 (5.06)
	WS scaled, X (SD)	10.79 (3.25)	12.46 (2.16)	11.11 (2.45)	9.09 (3.26)	8.55 (3.89)
	ELI composite, X (SD)	105.32 (19.27)	115.96 (10.61)	105.26 (9.72)	95.45 (18.60)	91.75 (27.43)

The goal of the work presented here is not to create a novel diagnostic test. Although the four tasks provide important information about a child's expressive language abilities, no developmental disorder, including DLD, is diagnosed on the basis of these four tasks alone. The purpose of the work here is to automatically score the four tests using computational methods and not to perform automated diagnosis or screening. We intentionally recruited participants with a range of diagnoses in order to ensure that these testing conditions were accessible to a wide range of children and abilities.

#### 2.1.2. Data Collection

Our goal was to demonstrate the technological capability to automate certain aspects of expressive language testing, not to create an automated version of a gold standard assessment. For this reason, we created parallel stimuli for our data collection designed to mimic the subtest objectives for a gold-standard assessment of expressive language. Each single-word stimulus was selected by an expert speech-language pathologist from a list of possible words matching the corresponding CELF-4 stimulus on age-of-acquisition (Kuperman et al., 2012), relative frequency (Masterson et al., 2003; Brysbaert and New, 2009; Davies, 2010), emotional valence (Mohammad and Turney, 2013), phonological/phonetic complexity, and overall appropriateness for the child language domain. Full sentence stimuli used identical or nearly-identical syntactic structures with each content word replaced by a word chosen by an SLP from a list of words matching the original content word on the above four dimensions. In addition, each sentence stimulus was matched to the original CELF-4 sentence in terms of its overall Flesch-Kincaid readability score (Kincaid et al., 1975) and its child language domain content. For example, many prompts in the original CELF-4 were specific to school, classrooms, and peer and family relationships, content that was mirrored in our stimulus selection. We then verified all stimuli via informal inspection of meaningfulness and appropriateness by all team members, including clinical experts in pediatric speech-language pathology, psychology, and psychiatry. We note that while that our versions of these subtests have not been subjected to the same scrutiny as the original CELF-4 stimuli and that they not been normed on a large population, the methodology we use to score a response given a stimulus, which is the focus on the work presented here, is independent of any particular stimulus.

The automated administration was conducted using a custom-built iPad application. The application presented the stimuli for four subtests, mimicking the original presentation prompts as closely as possible, and recorded the audio of children's spoken responses. The pacing and presentation of tasks and stimuli were controlled by a clinician using a second iPad. Trained examiners transcribed the responses and scored them according to conventional pen-and-paper rules.

#### 2.1.3. Expressive Language Tasks

Four subtests are included in this study: *expressive vocabulary* (EV), *word structure* (WS), *recalling sentences* (RS), and *formulated sentences* (FS). In the EV subtest, the child views an image and must verbally name the person, object, or activity depicted in that image. Responses are scored with full credit (2) if correct, partial credit (1) if the response is not incorrect but not specific enough (e.g., “fruit” for “lime”), and no credit (0) if the response is entirely incorrect. The test is discontinued if there are seven consecutive scores of zero.

WS is used to assess a child's grasp of inflectional morphology. A child is generally asked to complete a sentence after being given a prompt and shown a picture. Targets include morphemes expressing verb tense, possessives, plurals, and comparatives. An example item is, “the woman is fixing the car; here is the car that the woman… *[fixed]*.” Scores for this subtest are simply correct (1) or incorrect (0), and there is no discontinuation rule.

In the FS subtest, the child hears a target word and views a photograph of a scene, and must produce a complete sentence about the scene using the target word. The targets include a variety of word classes and increase in their syntactic requirements, including more challenging targets such as, “safely” and “because.” Responses are scored as 0,1, or 2, depending on the use of target word, grammatical correctness, and meaningful content. The test is discontinued if there are five consecutive scores of zero.

In the RS subtest, the child hears a sentence once (along with the video of a person saying the sentence in our automated task) and is asked to repeat it verbatim. Responses are scored on a scale of 0-3 based on number of errors (omissions, substitutions, transpositions). Repetitions are not counted as errors. The test administration is discontinued after five consecutive scores of zero.

#### 2.1.4. Data Scoring and Processing

During automated testing, examiners wrote down the child's response to the prompt. The audio recordings from the iPad application were later transcribed by research assistants, resulting in two transcripts for each audio response. We note that the content of these two transcripts often diverges. The response transcribed by the examiner in real time typically consists only of the word, phrase, or sentence to be scored, excluding any other commentary the child might provide. We refer to these transcripts as *clean transcripts*. The transcripts generated by the research assistants included all speech produced by the child that was recorded by the iPad, including comments and prefatory content. We refer to these transcripts as *verbatim transcripts*. When presented in the expressive vocabulary task with a picture of a pirate, a child might respond “I think that one's a pirate.” The verbatim transcript would include the entire utterance, while the clean transcript would include only the single target word “pirate.”

Responses were scored from the clean transcripts according to conventional scoring rules outlined briefly above in 2.1.3 and in detail in Semel et al. (2003). These rules include different stopping points (i.e., ceilings) for certain subtests and various rules for allowing partial credit on certain items. Further score calibration on each subtest then took place in consultation with a licensed SLP to increase scoring consistency and accuracy. Normative scores were calculated using the participants' ages at their first visit if they entered a new age bracket on the normative table during their participation. Study data were collected and managed using REDCap (Research Electronic Data Capture) (Harris et al., 2009), a secure, web-based application designed to support data capture for research studies. Clean data was processed through double-blind data entry, then evaluated for discrepancies utilizing REDCap electronic data capture tools. The process for one subtest, RS, differed in that clean transcripts were not available, so verbatim transcripts were manually reviewed (albeit without double-blind data entry) and edited to remove initial utterances (e.g., “um, I think it was…”) and repeated words, which are not counted as errors.

For each of the subtests, item scores are summed to create a raw score. These raw scores are then converted to norm-referenced scaled scores based on age and using a conventional look-up table. The scaled scores have an average of 10 with a standard deviation of three. Three of the subtests' scaled scores are summed (FS, RS, and WS), and using another look-up table, an Expressive Language Index (ELI) composite score is generated. This is a standard score with a mean of 100 and a standard deviation of 15. Please note that since the scaled scores and composite score are computed using the normative tables for the original CELF-4 test, these two score tiers must be recognized as approximations, but we include them to give a more clinically meaningful perspective to our scoring system.

### 2.2. Computational Models

#### 2.2.1. System Architecture

**Figure 1:**
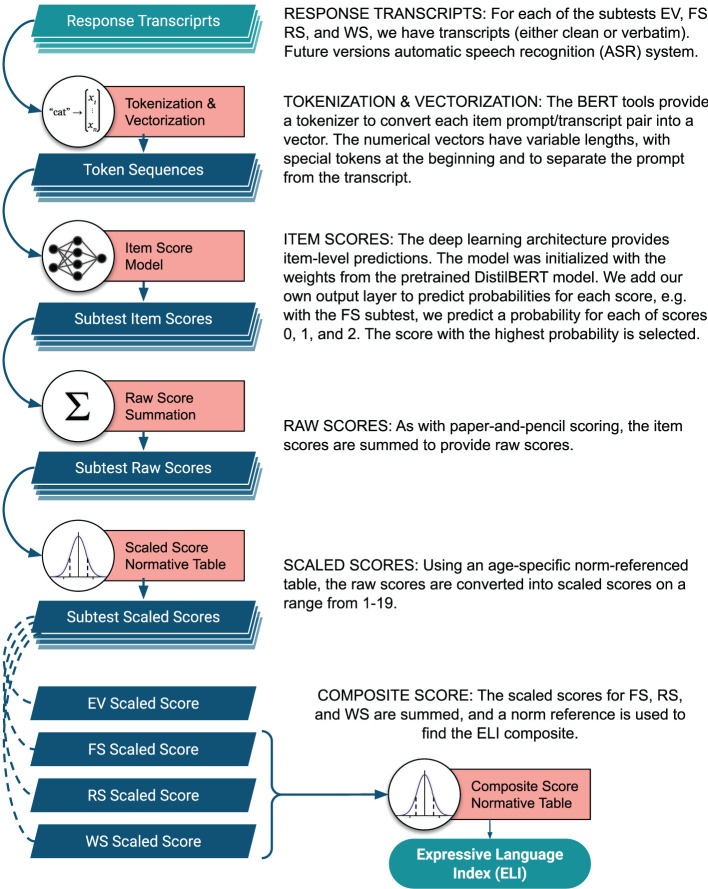
Diagram of the scoring system architecture.

Leveraging deep learning approaches, we develop a computational model that includes a modular, cohesive scoring system capable of producing item-level test scores directly from raw transcripts. The entire transcript (response word, phrase, or sentence) is tokenized and encoded into a variable-length vector, which is fed into a BERT (Bidirectional Encoder Representations from Transformers). BERT is a DNN-based language representation model that has achieved state-of-the-art performance in numerous downstream tasks such as sentiment analysis (Xie et al., 2020) and question-answering systems (Qu et al., 2019). Recent studies, including Chen et al. (2020) and our previous work on the FS subtest (Wang et al., 2020), have reported the superiority of such models in clinical tasks. The model has been fine-tuned to our task as described in section 2.2.2, and item-level scores are predicted for each subtest. Next, the model summarizes predicted item-level scores according to the scoring rules described in section 2.1.4 and sequentially computes raw score, scaled score, and ELI composite score across four subtests. A visual walk-through of the scoring system architecture is presented in Figure 1.

#### 2.2.2. Fine-Tuned BERT Classifier

The BERT model was originally trained on pairs of sentences to predict whether one sentence immediately followed the other in its original context, a task known as next sentence prediction, as well as a masked language modeling task in which the model uses the surrounding context to predict a word that has been removed from a sentence. Trained on a large corpus of unlabeled sentences—3.3 billion words from the English Wikipedia combined with BooksCorpus (Zhu et al., 2015)—language representations produced by BERT have been shown to be effective in capturing both syntactic and semantic aspect of the language (Ettinger, 2020; Rogers et al., 2020). Similar to the original BERT model, our task also involves pairs of “sentences”: prompts and responses. We form our input pairs to include the target word, phrase, or sentences alongside each response to provide the model with the same information a scorer would use, annotated with special BERT tokens to indicate breaks between prompt and response^1^. Given the prompt-response pairs along with manual scores (which serve as the gold standard), we develop four separate models to classify each stimulus response to EV, FS, RS, and WS subtest into scores of {0,1,2}, {0,1,2}, {0,1,2,3}, and {0,1}, respectively^2^.

We fine-tuned a variant of the original BERT model known as DistilBERT (Sanh et al., 2019) in our experiments. DistilBERT was specifically designed to reduce the number of parameters in the BERT model (66 vs. 110 M). Fewer parameters decreases the footprint of the model, and increases computation speed; more importantly, though, it decreases the risk of overfitting to the training data. We additionally added a L2 regularization term to further avoid overfitting^3^. A portion of the training data amounting to 10% of the entire data set was held out for validation. We fine-tuned the weights to the training data, and for every iteration over the entire training set (“epoch”), we also calculated the loss on the validation set^4^. When the validation loss failed to improve for four consecutive epochs, training was stopped, and the model was restored to the state with the best validation loss.

#### 2.2.3. Baseline Models

As a baseline for EV and WS scoring models, we developed support vector machine (SVM) classifiers trained on 300-dimensional vectors representing the mean semantic encoding of all the words in the response^5^. For the RS baseline model, we trained a SVM classifier on hand-crafted features that represent the correctness of a response. Inspired by our previous work (Gale et al., 2020), we trained an SVM classifier with correctness features extracted using Levenshtein edit distance to compute the frequency of errors (insertions, deletions, substitutions), as well as the number of correct words. In a variation of our previous work on the FS task (Wang et al., 2020), we developed a Multilayer Perceptron (MLP) classifier as baseline for FS using a sentence-level BERT embedding for each response. In contrast to our new models, this baseline model does not fine-tune the weights within BERT. Instead, only the MLP classifier is trained using the static embedding calculated by the pre-trained BERT model.

#### 2.2.4. Evaluation Metrics

To evaluate the performance of item-level classifiers, we computed the precision^6^, recall^7^, and classification accuracy^8^ for the item-level scores for each subtest. We further evaluated the predicted raw and scaled scores across sub-tests, in addition to the predicted composite score, using mean absolute error (MAE) between predicted and true scores. Each comprehensive scoring evaluation was performed using 5-fold cross-validation, shuffling the entire data set 20 times; we scored each repetition and present the average of the 20 scores^9^.

## 3. Results

For the evaluation, we compared the performance of our proposed models to baseline models using the evaluation metrics described in section 2.2.4. To explore the effect of extraneous words in transcripts, we examined our scoring models using our two input formats from section 2.1.4: clean (the scored response as annotated by trained examiners) and verbatim (which includes examiner speech and other superfluous chatter). Table 4 reports the accuracy of the baseline classifiers and our proposed BERT-based classifiers on each of the four subtests, separately trained and tested on clean and verbatim transcripts. Results, in the form of macro averages of 20 repetitions, indicate that the fine-tuned BERT models outperform baselines in terms of classification accuracy, precision, and recall across all four subtests. As expected, the clean transcripts are more reliably scored than the verbatim transcripts.

The fine-tuned BERT EV and WS models were the most accurate overall: EV had a 97% accuracy on verbatim transcripts, and both EV and WS had over 98% accuracy on clean transcripts. The FS scoring model showed the weakest performance, with the verbatim transcripts yielding 80% and 83% accuracy for the baseline and fine-tuned BERT models, respectively. This is not entirely surprising: FS responses are known to be difficult to manually score reliably, while the WS task is scored essentially on whether the child gives exactly the correct response. Full item-level classification results are shown in Table 2. We also present classification results for the BERT model as distinguished by diagnosis—typically developing (TD) vs. non-typically developing (non-TD)—in Table 3. The automatic scoring system scores the TD group more easily, with all measures consistently a few percent higher than the non-TD group.

**Table 2:** Mean (and standard deviation) of accuracy, precision, and recall measures for item-level scores.

		**Accuracy**		**Precision**		**Recall**	
**Subtest**	**Transcripts**	**Baseline**	**BERT**	**Baseline**	**BERT**	**Baseline**	**BERT**
EV	Clean	0.980 (0.001)	**0.986 (0.002)**	0.980 (0.001)	**0.986 (0.002)**	0.980 (0.001)	**0.986 (0.002)**
	Verbatim	0.939 (0.001)	**0.967 (0.002)**	0.941 (0.001)	**0.967 (0.002)**	0.939 (0.001)	**0.967 (0.002)**
FS	Clean	0.824 (0.019)	**0.856 (0.004)**	0.793 (0.015)	**0.842 (0.005)**	0.824 (0.019)	**0.856 (0.004)**
	Verbatim	0.801 (0.015)	**0.834 (0.006)**	0.759 (0.021)	**0.819 (0.005)**	0.801 (0.015)	**0.834 (0.006)**
RS	Clean	0.858 (0.001)	**0.872 (0.005)**	0.870 (0.001)	**0.873 (0.005)**	0.858 (0.001)	**0.872 (0.005)**
	Verbatim	0.846 (0.001)	**0.866 (0.005)**	0.854 (0.002)	**0.867 (0.005)**	0.846 (0.001)	**0.866 (0.005)**
WS	Clean	0.968 (0.001)	**0.984 (0.002)**	0.968 (0.001)	**0.984 (0.002)**	0.968 (0.001)	**0.984 (0.002)**
	Verbatim	0.940 (0.002)	**0.959 (0.002)**	0.939 (0.002)	**0.959 (0.002)**	0.940 (0.002)	**0.959 (0.002)**

*Mean values are the average over 20 repeats. Bold values in the tables were used to indicate the row's better score for each score type*.

**Table 3:** Distinguishing item-level scoring between typically developing (TD) and non-typically developing (non-TD).

		**Accuracy**		**Precision**		**Recall**	
**Subtest**	**Transcripts**	**TD**	**Non-TD**	**TD**	**Non-TD**	**TD**	**Non-TD**
EV	Clean	**0.990 (0.002)**	0.982 (0.003)	**0.990 (0.002)**	0.983 (0.003)	**0.990 (0.002)**	0.982 (0.003)
	Verbatim	**0.976 (0.003)**	0.960 (0.003)	**0.976 (0.003)**	0.960 (0.003)	**0.976 (0.003)**	0.960 (0.003)
FS	Clean	**0.869 (0.005)**	0.846 (0.006)	**0.854 (0.005)**	0.834 (0.006)	**0.869 (0.005)**	0.846 (0.006)
	Verbatim	**0.852 (0.006)**	0.820 (0.006)	**0.837 (0.004)**	0.805 (0.007)	**0.852 (0.006)**	0.820 (0.006)
RS	Clean	**0.886 (0.006)**	0.862 (0.007)	**0.887 (0.006)**	0.862 (0.007)	**0.886 (0.006)**	0.862 (0.007)
	Verbatim	**0.881 (0.006)**	0.855 (0.006)	**0.883 (0.007)**	0.855 (0.006)	**0.881 (0.006)**	0.855 (0.006)
WS	Clean	**0.993 (0.001)**	0.978 (0.003)	**0.993 (0.001)**	0.978 (0.003)	**0.993 (0.001)**	0.978 (0.003)
	Verbatim	**0.980 (0.002)**	0.944 (0.003)	**0.980 (0.002)**	0.943 (0.003)	**0.980 (0.002)**	0.944 (0.003)

*Mean (and standard deviation) of accuracy, precision, and recall measures for item-level scores. Mean values are the average over 20 repeats. Bold values in the tables were used to indicate the row's better score for each score type*.

**Figure 2:**
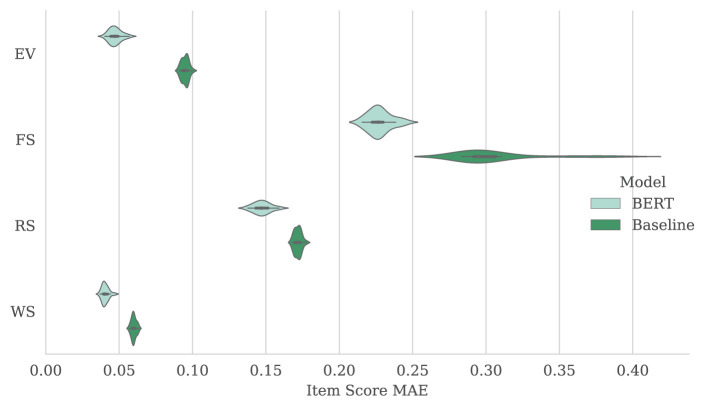
Mean (and standard deviation) of MAE results over 20 repeat experiments for item scores produced by baseline and fine-tuned BERT model. Results are shown using verbatim transcripts for each subtest (EV, FS, RS, and WS).

The fine-tuned BERT models outperformed baseline models in all cases, though as the scores were summed and normalized for raw and scaled scores, the difference in average MAE between models narrowed. Again, clean transcripts were more easily scored than their verbatim counterparts with the exception of the baseline RS model. Full score MAE results are shown in Table 4. Figure 2 shows the distribution of item score MAE on the verbatim transcripts for each subtest over 20 repetitions.

**Table 4:** Mean (and standard deviation) of mean absolute error (MAE) for score estimation at the item score, raw score, and scaled score tiers.

		**Item score MAE (std)**			**Raw score MAE (std)**			**Scaled score MAE (std)**		
		**Baseline**	**BERT**	**Scale**	**Baseline**	**BERT**	**Scale**	**Baseline**	**BERT**	**Scale**
EV	Clean	0.029 (0.001)	**0.020 (0.003)**	0–2	0.684 (0.041)	**0.500 (0.070)**	0–54	0.232 (0.019)	**0.144 (0.023)**	1–19
	Verbatim	0.095 (0.002)	**0.047 (0.004)**		1.807 (0.070)	**0.926 (0.093)**		0.647 (0.033)	**0.308 (0.033)**	
FS	Clean	0.262 (0.038)	**0.192 (0.007)**	0–2	3.747 (1.123)	**3.041 (0.258)**	0–48	1.421 (0.400)	**1.147 (0.099)**	1–19
	Verbatim	0.306 (0.029)	**0.228 (0.008)**		4.203 (0.877)	**3.339 (0.194)**		1.561 (0.303)	**1.254 (0.071)**	
RS	Clean	0.162 (0.002)	**0.140 (0.006)**	0–3	3.318 (0.078)	**2.225 (0.167)**	0–96	0.718 (0.031)	**0.488 (0.042)**	1–19
	Verbatim	0.172 (0.002)	**0.147 (0.006)**		2.575 (0.104)	**2.456 (0.208)**		0.562 (0.034)	**0.543 (0.058)**	
WS	Clean	0.032 (0.001)	**0.016 (0.002)**	0–1	0.817 (0.036)	**0.411 (0.059)**	0–32	0.508 (0.025)	**0.232 (0.040)**	1–19
	Verbatim	0.060 (0.002)	**0.041 (0.002)**		1.411 (0.063)	**0.926 (0.082)**		0.910 (0.046)	**0.574 (0.055)**	

*Results are presented for the baseline models from our previous work against the fine-tuned BERT models. Each of the EV, FS, RS, and WS subtests was scored automatically using clean and verbatim transcripts. Mean was calculated as a macro average over 20 repeats. Bold values in the tables were used to indicate the row's better score for each score type*.

Aggregating the FS, RS, and WS scores to approximate ELI composite scores, the baseline was substantially outperformed by the fine-tuned BERT model when using clean transcripts, with an MAE of 3.405 and 3.166 points, respectively (on a scale of 45–155). Performance of the two models was more comparable when using verbatim transcripts, with an MAE of 4.266 and 3.709 points for the baseline and fine-tuned BERT models, respectively. These results are presented in Table 5, with the distribution over 20 repetitions also illustrated in Figure 2.

**Table 5:** Mean absolute error (MAE) and standard deviation for expressive language index (ELI) composite score with lookup table applied to scaled score estimation.

			**ELI composite MAE**		**ELI composite MAE**		**ELI composite MAE**	
			**TD and Non-TD**		**Non-TD**		**TD**	
**Subtests**	**Transcripts**	**Scale**	**Baseline**	**BERT**	**Baseline**	**BERT**	**Baseline**	**BERT**
FS, RS, WS	Clean	45–155	3.405 (0.703)	**3.166 (0.235)**	3.863 (0.906)	**3.507 (0.304)**	2.809 (0.471)	**2.723 (0.206)**
	Verbatim		4.266 (0.601)	**3.709 (0.231)**	5.030 (0.704)	**4.144 (0.351)**	3.273 (0.514)	**3.144 (0.184)**

*Results are split out for typically developing (TD) and non-typically developing (non-TD) participants, as well as the combined population. Bold values in the tables were used to indicate the row's better score for each score type*.

**Figure 3:**
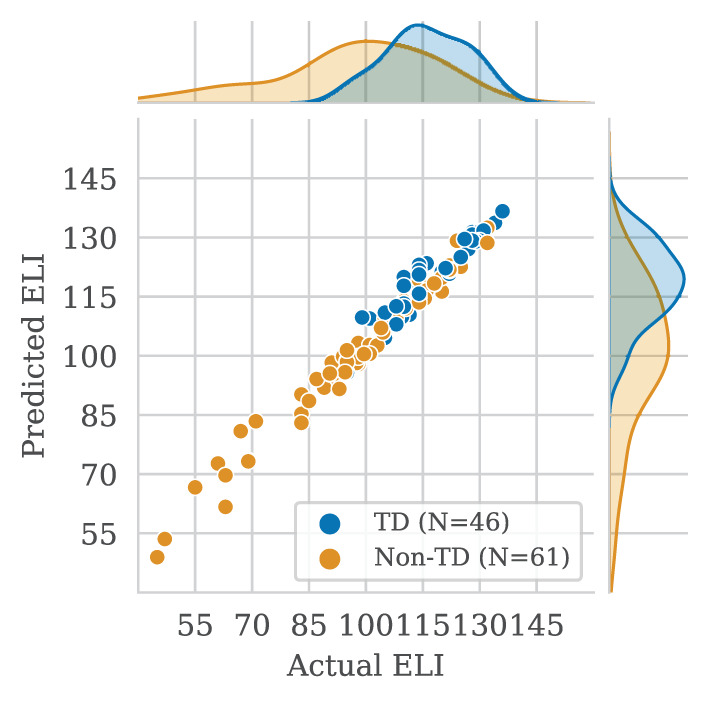
Correlations of actual vs. automatically predicted Expressive Language Index (ELI) composite scores. Distributions are shown for participants with and without developmental language disorder (DLD). Each point represents the average scores for one participant.

Lastly, we present Spearman's correlations for predicted vs. true scores in Table 6. The fine-tuned BERT model outperformed the baseline in nearly all configurations. Raw scores for EV, RS, and WS were among the highest at about 99%. The lowest correlation was in FS item scores: the baseline showed 73.0% and 67.6%, while the fine-tuned BERT model had 80.4% and 76.3% for clean and verbose transcripts, respectively. Overall, ELI composite scores correlated highly. The fine-tuned BERT model had correlations of 98.0% and 96.9% for clean and verbose transcripts, respectively. In the RS subtest, the baseline model's raw and scaled score correlations were higher than the BERT model, though the difference was negligible. In Figure 3 we illustrate correlations between predicted and true ELI composites, distinguishing between TD and non-TD participants.

**Table 6:** Average Spearman's correlation results over 20 repeats (with standard deviations) for Item, Raw, and Scaled Score estimation.

		**Item** **ρ**		**Raw** **ρ**		**Scaled** **ρ**		**ELI** **ρ**	
**Subtest**	**Transcript**	**Baseline**	**BERT**	**Baseline**	**BERT**	**Baseline**	**BERT**	**Baseline**	**BERT**
EV	Clean	0.971 (0.002)	**0.981 (0.003)**	0.987 (0.002)	**0.990 (0.003)**	0.980 (0.002)	**0.989 (0.003)**		
	Verbatim	0.894 (0.003)	**0.952 (0.005)**	0.939 (0.005)	**0.978 (0.004)**	0.916 (0.007)	**0.971 (0.006)**		
FS	Clean	0.730 (0.045)	**0.804 (0.007)**	0.857 (0.122)	**0.924 (0.012)**	0.815 (0.122)	**0.886 (0.017)**		
	Verbatim	0.676 (0.035)	**0.763 (0.010)**	0.831 (0.102)	**0.913 (0.015)**	0.769 (0.097)	**0.854 (0.019)**		
RS	Clean	0.943 (0.001)	**0.951 (0.002)**	**0.987 (0.001)**	0.986 (0.001)	**0.978 (0.002)**	0.977 (0.002)	0.969 (0.020)	**0.980 (0.002)**
	Verbatim	0.937 (0.001)	**0.948 (0.002)**	**0.987 (0.001)**	0.986 (0.001)	**0.978 (0.002)**	0.976 (0.003)	0.956 (0.018)	**0.969 (0.003)**
WS	Clean	0.888 (0.004)	**0.946 (0.006)**	0.975 (0.002)	**0.992 (0.002)**	0.953 (0.004)	**0.988 (0.003)**		
	Verbatim	0.786 (0.006)	**0.860 (0.009)**	0.940 (0.006)	**0.970 (0.004)**	0.907 (0.010)	**0.965 (0.005)**		

*For all values, p < 00.001. Bold values in the tables were used to indicate the row's better score for each score type*.

## 4. Discussion

In this paper, we describe a set of highly accurate computational models developed for scoring responses to several expressive language tasks for children. The models were combined into a comprehensive, multi-level scoring system based on conventional scoring methods. We automatically produced an item score for each subtest prompt, as well as a raw total score and a norm-referenced scaled score for each subtest. Three of the four subtests contribute to a composite score called the Expressive Language Index, which we also computed. The very high levels of accuracy of our results on several dimensions point to the utility and potential of automated computationally-driven methods of both administering and scoring expressive language tasks for pediatric developmental language evaluation.

### 4.1. Clinical Significance

While we would like to remind the reader that our scaled scores and composite score are approximations (as discussed in section 2.1.4), we believe these results in particular show promise for clinical applications. Our approach relies on a fine-tuned BERT modeling system which produced improved scores in all configurations. Even in the FS task, the most challenging subtest to score, the MAE of the scaled score, which ranges from 1 to 19, with a mean of 10 and standard deviation of 3, is just over 1 point. The CELF-4 manual considers a subtest scaled score below 7 points to be suggestive of clinical concern. An automated system that can produce scaled scores within about a point of the expert-derived score is likely to be clinically valuable. The system's best accuracy for scaled score prediction was for the EV subtest, a confrontation naming task. Our new model has an MAE of well under half a point on verbatim transcripts, which demonstrates the potential of fully automated scoring of the EV task. Accuracy for this type of testing is particularly encouraging, as there are long used standalone language assessments with this focus (e.g. Kaplan et al., 1983; Martin and Brownell, 2011; Roth, 2011). Automation of this type of language assessment could have great clinical relevance. Lastly, the ELI composite score is predicted within 4 points on the standard score scale of 45–155, generally well within the score's confidence interval.

### 4.2. Comparison With Prior Work

The baseline models represent our previous best efforts in scoring each of the four subtests. The fine-tuned BERT methods presented here provide many natural advantages. Some of these advantages might explain the improvements over the baselines that we observe here, while others may hold promise for future advances in scoring, as well as deeper insight.

Our baseline RS model relied on explicitly engineered features and inherently discarded valuable information. For example, the Levenshtein method of error detection is unable to distinguish between an insertion and the superfluous chatter that distinguished our verbatim transcripts from the clean ones. The Lenvenshtein approach to scoring would be unable to distinguish between a common developmental error from a misrecognition in transcripts generated via automatic speech recognition. In addition, there may be latent clinically relevant information in responses that can be captured using contextualized language representation models like BERT but that would not easily be captured via rule-based scoring. The structure of BERT models extends well to introspective analysis, with techniques and tools for word-relationship heat maps and other such visualizations (Hanselowski and Gurevych, 2019; Kovaleva et al., 2019; Wu et al., 2020).

For the EV and WS models, we used word2vec embedding models, which do not effectively capture contextual information and typically rely on sums or averages of individual word vectors. In addition, the pre-trained word2vec model we used in our baselines and the fixed DistilBERT model used with FS were both trained on texts written by adults and were not fine-tuned to our domain of child language. Fine-tuning the weights of the DistilBERT model allows us to take advantage of the broad representation abilities of a pre-trained language model while adapting it to the idiosyncratic language of our scoring task.

Another motivation for using the fine-tuned BERT model was the prospect of a unified approach to scoring different subtests. Although the content and scoring rules differ across the four subtests, our new system relies on a single architecture and differs only in terms of the data used to train the models. As a result, we benefit from a single pipeline for data preparation and feature engineering, and any improvements we make to the pipeline and architecture are more likely to apply to all subtests.

### 4.3. Future Work

Our vision for a fully automated scoring system starts with a spoken response. We intend to bring automatic speech recognition (ASR) into our system pipeline to automatically convert a participant's recorded voice into written transcripts. The results presented here demonstrate how our fine-tuned BERT model adapts to the extraneous information present in verbatim transcripts. ASR transcripts contain the same extraneous information, and we expect a 10–20% word error rate on top of that (Gale et al., 2019; Wu et al., 2019). Our previous experiments with the RS subtest (Gale et al., 2020) demonstrate the resilience of automated scoring when challenged with ASR transcripts, and we expect that our improved models may be even better equipped to adapt to ASR transcripts.

Our models could benefit from more (and more varied) training samples, but the time and effort required for test administration, transcription, and scoring makes it difficult to procure more data. Our previous work in Wang et al. (2020) used machine translation technology to “translate” correct responses into partial- and no-credit responses, which was used as artificial data during training. That work showed how augmenting the training data with the artificial responses improved the performance of scoring models. We intend to incorporate this data augmentation technique into our new FS model, as well as adapt the technique to the other three subtests.

### 4.4. Conclusions

Even before the pandemic, many communities lacked regular access to clinicians and practitioners trained in assessing child language. The restrictions on travel and facility capacity that most of the world is now experiencing have further exacerbated this inequitable access to patient care. Methods of computerized administration and automatic scoring of language assessment instruments have the potential to reach underserved populations and to enable speech-language pathologists to devote more time to developing and applying interventions and treatments. Although it is certainly not the case that an automated system will ever be an adequate or comparable replacement for an expert clinician, technologies like the one proposed here can provide crucial support for these experts and for the schools and families that they serve.

## Data Availability Statement

The raw data supporting the conclusions of this article will be made available by the authors, without undue reservation.

## Ethics Statement

The studies involving human participants were reviewed and approved by OHSU Institutional Review Board. Written informed consent to participate in this study was provided by the participants' legal guardian/next of kin.

## Author Contributions

MA, JD, JB, JS, and EP contributed to the conception, design, and implementation of the data collection. RG designed, implemented, and evaluated the automated scoring system, with contributions from EP and YW. EP, JD, MA, and RG jointly wrote the manuscript and all authors read and approved the submitted version.

## Conflict of Interest

The authors declare that the research was conducted in the absence of any commercial or financial relationships that could be construed as a potential conflict of interest.

## Footnotes

^1^For example, if an EV prompt was a picture of a spatula, and the child responded “pancake turner,” the sequence would be provided to the model as “[CLS] spatula [SEP] pancake turner [SEP]”.

^2^We replaced the original output layer of BERT (a binary classifier) with a single softmax layer of *k* nodes, predicting one likelihood for each item-level score of 0–*k*).

^3^L2 regularization was performed with the constant weight of 0.001.

^4^We trained the model with the learning rate of 0.00001 on batches of 16 response sequences at a time, targeting a categorical cross-entropy loss.

^5^Word vectors were computed using the pre-trained *spacY* word2vec (Mikolov et al., 2013; Honnibal et al., 2020) model “en_core_web_lg,” an embedding model trained on a large corpus of web-based English text.

^6^Precision $=\frac{\text{TP}}{\text{TP}+\text{FP}}$, where TP and FP are true positives and false positives, respectively. We present precision as a weighted average of each class's score.

^7^Recall $=\frac{\text{TP}}{\text{TP}+\text{FN}}$, where TP and FN are true positives and false negatives, respectively. Note that is the same measure as sensitivity but without the assumption that we are limited to two classes. We present recall as a weighted average of each class's score.

^8^Accuracy $=\frac{\text{TP}+\text{TN}}{\text{A}}$, where TP, TN, and A are true positives, true negatives, and the entire population, respectively.

^9^To validate results and establish their independence from our specific data sets, and also to reduce the overfitting problem, we used cross-validation techniques in which the training, development, and test sets are rotated over the entire data set. With this approach, the optimal parameters of the models were learned only from the training examples and never from the examples used to test the model.
